# Utilizing multi-criteria decision-making analysis and 3D visualization techniques for dam site selection and irrigation area identification in Gedeb River, Ethiopia

**DOI:** 10.1016/j.heliyon.2024.e35604

**Published:** 2024-08-02

**Authors:** Fekadu Temesgen, Baye Terefe

**Affiliations:** a Department of Remote Sensing, SSGI, Addis Ababa University, Addis Ababa ,Ethiopia; b Department of Geography and Environmental Studies, Injibara University, Injibara , Ethiopia

**Keywords:** Dam site selection, Gedeb river, Irrigation area, 3D visualization, Multi-criteria decision-making

## Abstract

Irrigation dams and irrigation suitability analysis is important for optimal water management, crop selection and productivity, water conservation, environmental sustainability, and economic viability in agriculture arena. Thus, the main objectives of this study were to identify a suitable dam site and irrigation area in the Gedeb River, Ethiopia, using Multi-Criteria Decision-Making analysis and 3D Visualization techniques. To identify a suitable dam site, various parametrs such as rainfall, runoff, stream flow, mineral site, faulting areas, landslide site, rock types, elevation points, relief features, soil types were used while to identify a suitable irrigation area, different parametrs such as altitude, slope, soil, geological structure, distance, and land use land cover datasets were used. The necessary dataset which were used to identify a suitable dam site and irrigation area collected from Ethiopian Mapping Authority (EMA), Ethiopian irrigation and energy ministry freely. In addition, for the final irrigation dam site selection and suitable irrigation area in the Gedeb watershed, multi-criteria decision-making method with expert judgment were applied respectively. Based on the study's findings, a suitable irrigation water reservoir dam covering an area of 1886 ha, with a potential water holding capacity of 2,961,145,697 cubic meters was identified. The results also revealed a highly suitable area of 18,362.05 ha, a moderately suitable area of 19,204.05 ha, a marginally suitable area of 2095.25 ha, and a not suitable area of 2.89 ha for the aforementioned purpose. The methodological approach and research findings presented in this study can greatly assist government and non-governmental organization planners and decision-makers in the development of irrigation projects.

## Article Highlights

• Identified suitable dam sites using advanced methods in Gedeb River, Ethiopia.
• Determined irrigable areas based on factors of suitability analysis.
• Proposed reservoir with 2.9 billion cubic meters capacity for irrigation.

## 1 Introduction

Irrigation dams are essential for water management, agricultural development, flood control and ensuring water availability for various sectors [[1], [2], [3], [4]]. Thus, it is imperative to choose a suitable location for an irrigation dam for multiple reasons [5]. A strategically designed site maximises agricultural productivity by guaranteeing ideal water distribution, storage, and supply to the designated locations [4]. Secondly, environmental considerations should be taken into account during the site selection process to reduce any potential harm to nearby communities and ecosystems [6]. Last but not least, selecting the ideal location can aid in reducing possible hazards including soil erosion, flooding, and sedimentation [7]. The process of choosing a location for irrigation dams is intricate and multidimensional, requiring a thorough comprehension of hydrological, environmental, geotechnical, and socioeconomic variables [8,9]. A strategically chosen dam location can optimise agricultural yield, guarantee sustainable water management, and reduce adverse effects on the surrounding ecosystem and communities [10,11]. In addition, irrigation suitability analysis is important for optimal water management, crop selection and productivity, water conservation, environmental sustainability, and economic viability [12]. It provides valuable insights that enable stakeholders to make informed decisions and ensure the sustainable and efficient use of water resources in agriculture [13].

Identifying suitable irrigation dam sites and irrigation areas in a specific region involves a comprehensive assessment of conducting a feasibility study, assessing water resources, analysing topography and geology, evaluating soil suitability, considering the environmental impact, analysing socio-economic factors, using GIS and remote sensing techniques, conduct a cost-benefit analysis and consult experts and stakeholders [14,15]. By following these steps and considering the relevant factors and decision-making process, suitable irrigation dam sites and irrigation areas in a specific region can be identified [16]. In this situation, the identification of irrigation regions and the selection of dam sites are crucial tasks for multi-criteria decision-making (MCDM) systems [[17], [18], [19]].By simplifying the evaluation of numerous criteria, weighting and ranking criteria, analysing trade-offs, offering decision support, and encouraging transparency and stakeholder involvement, the methodologies help with the selection of dam sites and irrigation areas [20,21]. These methods improve the decision-making process by guaranteeing that the irrigation area and dam location are selected to suit the needs of the particular area and the intended goals.

Moreover, after the crucial irrigation dam site is selected and a suitable irrigation area is identified, planners and designers use 3D modelling techniques to portray accurate visualization [22]. 3D modelling is necessary for the site preparation of irrigation dams and irrigation area identification and provides accurate visualization, improves design and analysis capabilities, enhances communication and collaboration, saves costs and time, and supports safety and risk assessment [23]. It is a valuable tool that can significantly contribute to successfully planning and implementing irrigation dam projects. Therefore, the 10.13039/100031019Digital 10.13039/100028488Elevation Model (DEM) is a necessary source of remote sensing data and is used to show accurate visualization in 3D modelling as it provides detailed elevation data, enables realistic representation of the terrain, supports precise spatial analysis, facilitates hydrological modelling, and allow for integration with other geospatial data [24,25]. By incorporating DEM into the 3D modelling process, engineers and planners can create more accurate and reliable visualizations of the dam site, important to well-informed decision and improved project outcomes [26]. In addition, DEM is a fundamental authoritative source of remote sensing data [[27], [28], [29]] and one of the primary layers of spatial data infrastructure in national, regional, and continental topographic databases [30,31]. The quantitative characterization of land surface configuration is becoming increasingly popular as a method of modelling the interactions of numerous terrestrial dynamic systems, including geologic, geomorphic, and hydrologic processes [[32], [33], [34]]. This better reality has increased the demand for large-area DEMs such as TanDEM-X, MERITDEM, ALOS Parcel, and ASTER DEM satellite altimeter missions [35,36]. The proliferation of DEMs for topographic research has allowed for a more in-depth exploration of the relationship between geomorphic processes and landforms as well as improved height 3D spatial modelling [37]. Digital Elevation data have many applications in GIS and are the most common basis for 3D spatial modelling [38,39]. DEMs are integral elements for mapping, geospatial visualization, the generation of orthophoto and are also used for 3D analysis in different areas such as environmental modelling and hydrology analysis [[40], [41], [42]].

Furthermore, in the case of hydrological modelling [43], using DEM, it is possible to extract information about the topographic features' ability to accumulate surface water for irrigation site selection for better 3D visualization [44,45], because 3D surface modelling visualization has become one of the leading architectural abilities to visualize topographic features in 3D spatial view [[46], [47], [48]]. In this regard, if DEM products can display topographic elements in a readily recognized 3D spatial view, it will be extremely useful to monitor and select any landscape for any purpose [49]. Thus, using DEMs, 3D spatial modelling water-related conservation, and irrigable agricultural area projects have to be applied everywhere and can increase agricultural production using irrigation activities [[50], [51], [52], [53]]. A human-made water reservoir dam modelled by 3D spatial modelling using DEM data is one of the water-related conservation methods that are used and help to collect surface water in the world today [54,55]. It can maximize agricultural production and stabilize the local market in advance [56]. Along with this, one of the basic means of increasing global agricultural production and sustaining food security and livelihood of society is constructing irrigation dams in suitable agricultural areas using 3D spatial modelling techniques by DEMs data, which can be functional [[57], [58], [59]] if such sites are selected through scientific methods of 3D spatial modelling site selection techniques using DEMs data for better visualization [60].

Therefore, the modern technology and research development enhance and deliver optimized irrigation potential areas concerning suitable dam construction site selection for water resource reservoirs using a 3D spatial modelling system by DEMs data [61,62]. Finally, the potential irrigation areas concerning suitable dam construction site selection for water resource reservoirs of an area can be functional when the project plan is supported properly and portrayed in 3D spatial modelling using DEM data for better visual impression [63,64]. The evaluation of appropriate dam construction site selection for water reservoirs and land suitability for irrigation is crucial for designing and implementing irrigation projects and boosting agricultural production [65].

However, various studies have been conducted regarding the irrigation dam site selection [21,42,44,61,62,[66], [67], [68], [69]] and other many more investigations. But, these studies have limited merly on irrigation dam site selection; and could not address irrigation area identification after the dam site selection in their study results. In addition, different studies have been executed regarding the irrigation suitability analysis [14,16,19,[70], [71], [72], [73], [74]] and other many more studies. However, these studies have limited only on irrigation area identification; and could not address suitable irrigation dam site selection in their study findings. Generally, the aforementioned studies could not show the suitable irrigation dam site selection and irrigation site identification together. Moreover, even though the bottom part of the Gedeb watershed has a suitable potential area for a water reservoir and irrigable agricultural field, which can increase agricultural production for the surrounding community, but both the community and government are not well informed about these potential areas. Furthermore, no research could be undertaken in this area to point out these novel ideas. Following these perspectives, the main objective of this study is to identify a suitable potential site for a water reservoir and irrigable agricultural field using various determinant factors and showing the result with 3D spatial modelling techniques.

Overall, this study result provides valuable guidance for water resource utilization and management, land-use management, soil conservation management in regions where naturaly endowed by water resource, suitable irrigation damsites and suitable irrigation areas. In addition, focusing merly the Jedeb river dewellers in consideration, the study result offers a valuable framework for planning and implementation of local area irrigation projects, ultimately enhancing the surrounding communities agricultural production and supporting the livelihood of the local communities.

## 2 Material and methods

### 2.1 Description of the study area

**Fig. 1:**
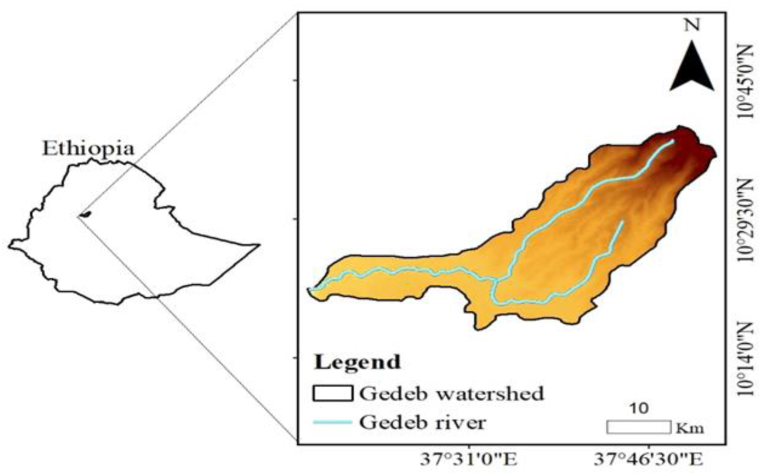
Location map of the Gedeb watershed.

The study area is located between 10°17′12.646″N to 10°40′7.451″N and 37°16′58.461″E to 37°52′12.397″E (Fig. 1). The Elevation of the study area ranges from 1498 to 3990 m.a.s.l. The minimum and maximum mean annual air temperatures in the area are 10.3 °C and 20.1 °C, respectively. In the study area, the average annual rainfall ranges from 900 to 1800 mm. The Gedeb watershed's principal soil types are leptosols, nitosols, vertisols, and cambisols. Leptosols exist in the upper and lower parts of the watershed, covering 22.89 km^2^, while Nitosols appear in the central section of the watershed, covering 197.13 km^2^. Furthermore, Vertisols are found across the watershed, covering an area of 778.05 km^2^, while Cambisols are found in the watershed's pocket, which covers 8.96 km^2^. Farmland/settlement, grassland, forest land, and barren land cover 242.68 km^2^, 350.76 km^2^, 198.09 km^2^, and 215.104 km^2^ of the Gedeb watershed, respectively.

### 2.2 Data sources and methods of collection

To run this study, different secondary data were used. The necessary secondary data sets were used to accomplish this task (Table 1).

**Table 1:** Data sources.

Data set types	Format	Resolution	Data source	Derived maps/items
SPOT imagery	Raster	0.5m	EMA	LULC map
ASTER DEM	Raster	20m	USGS	Slope, altitude, distance
Stream flow	Numeric	–	Ministry of EI	Historical stream flow
Runoff	Numeric	–	Ministry of EI	Historical runoff
Rainfall	Numeric	–	Ministry of EI	Historical rainfall condition
Soil	Vector		EMA	Soil map
Rock type	Vector	–	EMA	Rock map
Mineral type	Vector	–	EMA	Mineral type map
Relief features	Vector	–	EMA	Elevation map
Faulting Areas	Vector	–	EMA	Faulting map
Landslide location	Vector	–	EMA	Landslide map
Geology	Raster	–	EMA	Geological map
Dam crest GCPs	vector	–	Author	Dam crest points
River bed GCP	Vector	–	Author	Dam bed point

### 2.3 Methods of data analysis and interpretation

#### 2.3.1 Irrigation dam site selection factors

The following key considerations were taken for irrigation dam site selection in the study.

##### 2.3.1.1 Hydrological factors

**Fig. 2:**
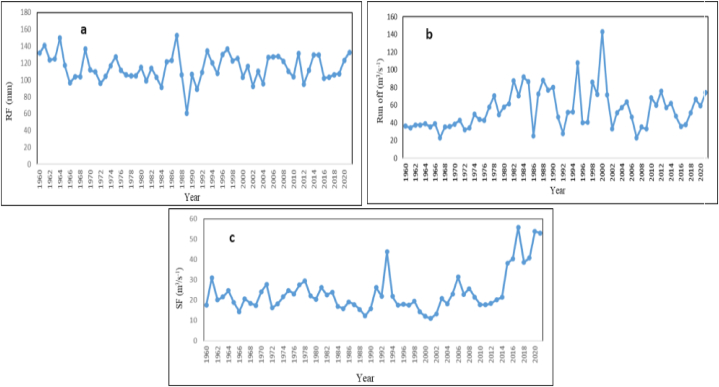
Gedeb river trends of average annual rainfall (a), runoff (b) and stream flow (c) (1960–2021).

**Rainfall:** during the 1960–1987 periods, the average annual rainfall of the Gedeb River followed increasing trends and it increased from 123 mm in 1960 to 153 mm in 1987, an annual increase of 1.11 mm. In contrast; from 1987 to 1989 the average annual rainfall showed decreasing trends and it decreased from 153 mm in 1987 to 60 mm in 1989, an annual decrease of 46.5 mm. But, again from 1989 to 2021 the average annual rainfall portrayed increasing trends and it increased from 60 mm in 1989 to 133 mm in 2021, an annual average increase of 2.25 mm. Generally, the temporal distribution of the average annual rainfall of the Gedeb River in the period between 1960 and 2021 showed a consistently increasing trend from the amount between 132 mm and 133 mm (Fig. 2a). Thus, the watershed has reliable, sufficient and regular rainfall trends and it meets the irrigation dam site demands.

**Watershed runoff:** during the 1960–2000 periods, the average annual runoff of the Gedeb watershed followed increasing trends and it increased from 36 m^3^s^-1^ in 1960 to 143 m^3^s^-1^ in 2000, an annual increase of 2.675 m^3^s^-1^. In contrast, from 2000 to 2002 the average annual run-off showed decreasing trends and it decreased from 143 m^3^s^-1^ in 2000 to 33 m^3^s^-1^ in 2002, an annual decrease of 55 m^3^s^-1^. But, again from 2002 to 2021 the average annual run-off portrayed increasing trends and it increased from 33 m^3^s^-1^ in 2002 to 74 m^3^s^-1^ in 2021, an annual average increase of 2.16 m^3^s^-1^. Generally, the temporal distribution of the average annual runoff of the Gedeb River in the period between 1960 and 2021 showed a consistently increasing trend from the amount between 36 m^3^s^-1^ to 73 m3s-1 (Fig. 2b). Thus, the watershed has a reliable, sufficient and regular runoff trend. So, it meets the irrigation dam site demands.

**Stream Flow:** during the 1960–1993 periods, the average annual stream flow of the Gedeb River followed increasing trends and it increased from 18 m^3^s^-1^ in 1960 to 44 m^3^s^-1^ in 1993, an annual increase of 0.78 m^3^s^-1^. In contrast; from 1993 to 2001 the average annual stream flow showed decreasing trends and it decreased from 44 m^3^s^-1^ in 1993 to 11 m^3^s^-1^ in 2001, an annual decrease of 4.125 m^3^s^-1^. But, again from 2001 to 2006 the average annual stream flow portrayed increasing trends and it increased from 11 m^3^s^-1^ in 2001 to 31 m^3^s^-1^ in 2006, an annual average increase of 4 m^3^s^-1^. In contrast, from 2006 to 2014 periods, the average annual stream flow followed decreasing trends. It decreased from 31 m3s to 1 in 2006 to 21 m^3^s^-1^ in 2014, an annual decrease of 1.25 m^3^s^-1^. In contrast, the annual average stream flow of the target area followed increasing trends and it increased from 21 m^3^s^-1^ in 2014 to 53 m^3^s^-1^ in 2021, an annual average increase of 4.57 m^3^s^-1^. Generally, the temporal distribution of the average annual stream flow of the Gedeb River in the period between 1960 and 2021 showed a consistently increasing trend from the amount between 18 m^3^s^-1^ to 53 m3s-1 (Fig. 2c). Thus, the watershed has a reliable, sufficient and regular-stream flow trend. So, it meets the irrigation demands.

**Catchment area:** The Gedeb watershed is one of the largest watersheds in Ethiopia and it covers a total area of 100667.57ha. The watershed has many small tributaries and one big river which flows regularly throughout the year. The catchment area has four different seasons (Summer, Winter, Spring and Autumn). Summer is the main rainy season in the watershed. Generally, taking into account the watershed characteristics, the target area has the best water inflow, distribution efficiency and storage capacity throughout the year. Thus, the watershed has reliable and sufficient water sources to meet the irrigation dam site demands.

##### 2.3.1.2 Geotechnical considerations

**Fig. 3:**
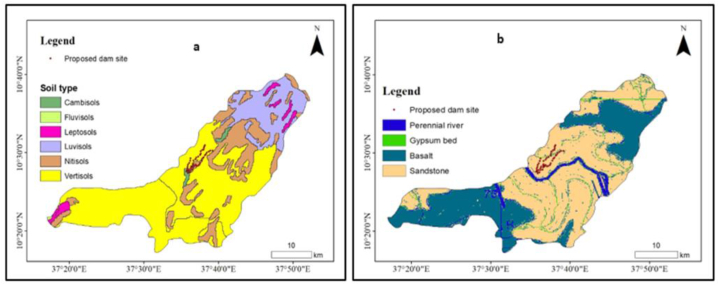
Soil type (a) and rock type (b) in the Gedeb watershed.

**Soil types:** the soil composition should be suitable for dam construction and able to withstand the weight of water. Taking this idea into account, the Gedeb watershed has cambisols, fluvisols, leptosols, luvisols, nitisols and vertisols and from which the proposed irrigation dam site is found in the vertisols (Fig. 3a). The vertisols have a high water retention capacity. Thus, the proposed irrigation dam site has a high water logging capacity. So, it meets the irrigation dam site demands.

**Rock types:** the gypsum bed, basal and sandstone three major rock types are found in the Gedeb watershed and the proposed irrigation dam site is located in the sandstone rock types (Fig. 3b). The sandstone has the characteristic of being cemented firmly by a slowly weathered mineral and resists mechanical breakdown. Thus, the proposed irrigation dam site will have a high load-bearing capacity. So, it meets the irrigation dam site demands.

**Fig. 4:**
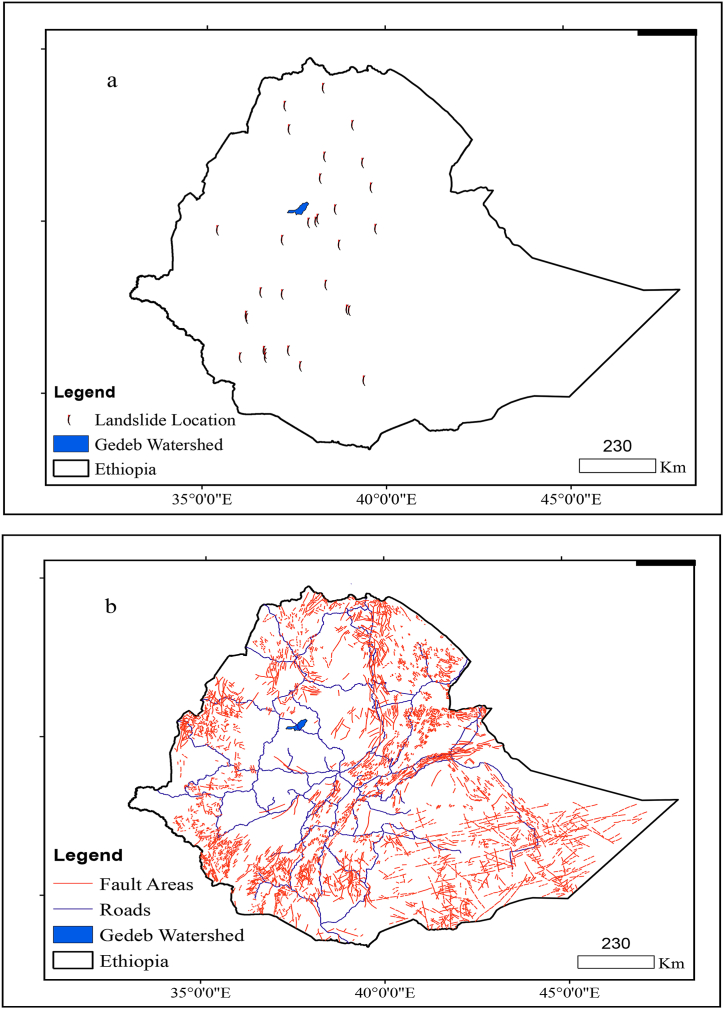
Landslide location (a) and Fault areas (b) in Ethiopia.

**Geological conditions:** Landslides are found from the northern to southern central parts of Ethiopia. Even though the landslides are located in different parts of the nation, the Gedeb watershed is free from any landsides. Thus, the watershed is free from any landslides and it meets the irrigation dam site demands (Fig. 4a). Similarly, faults are found everywhere in Ethiopia. Even though the faults are located in different parts of the nation, the Gedeb watershed is free from any faults. Thus, the watershed is free from any faults and it meets the irrigation dam site demands (Fig. 4b).

**Fig. 5:**
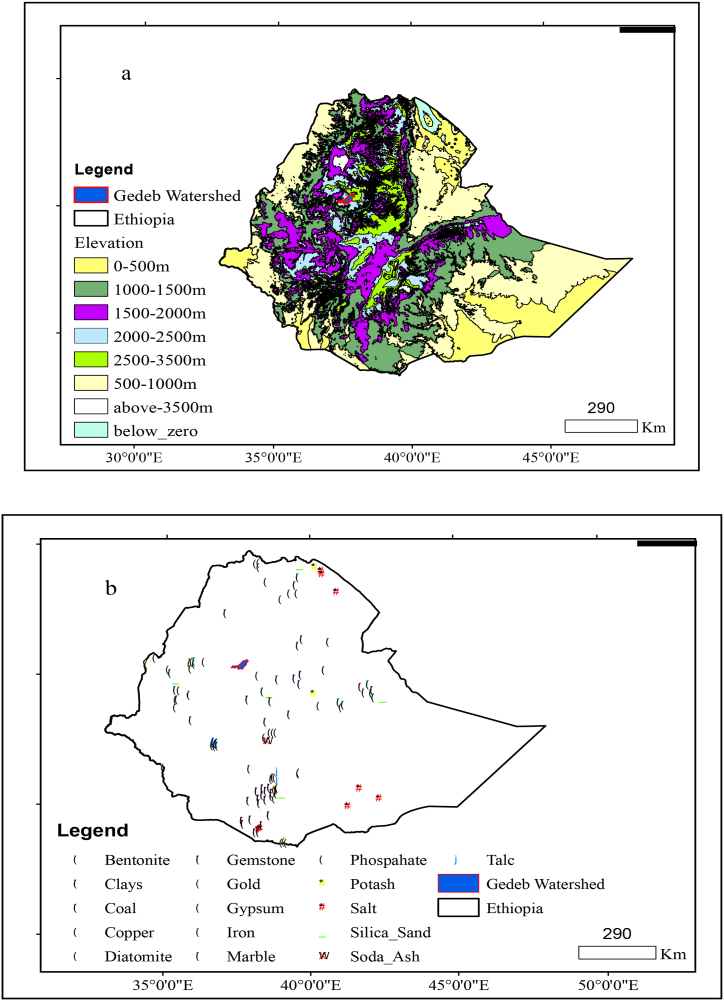
Relief features (a) and mineral types (b) of Ethiopia.

Moreover, Ethiopia has diverse elevation classification which ranges from the highest 3500m above sea level to and lowest zero meters below sea level. In this situation, the Gedeb watershed is found between 2000 and 3500 m above sea level. Thus, the watershed is free from any water source scarcity and drought problems (Fig. 5a) and it meets the irrigation dam site demands. Furthermore, Ethiopia has different industrial minerals which are located in various parts of the nation but the Gedeb watershed is free from any industrial minerals (Fig. 5b). Thus, the target area does not any national side effects about industrial minerals extravagancy and it meets the irrigation dam site demands.

##### 2.3.1.3 Environmental impact

**Ecological balance:** the irrigation dam site selection process should consider potential impacts on flora, fauna, and aquatic ecosystems scientifically [75]. The proposed irrigation dam site is found following the Gedeb River low laying and in this place, there are no aquatic ecosystems. This river bed consists of very dwarf diverse flora which can not give any service. In addition, following these diverse flora, various bird species are also found. Even though the area has such an ecological relationship, the irrigation dam benefits outweigh the impacts. Thus, the target area does not have such a credential side effect on environmental impacts and it meets the irrigation dam site demands.

**Social and cultural aspects:** The irrigation dam site selection should not adversely affect local communities, their livelihoods, or cultural heritage sites [76]. The local community produce commercial food crops like potato, tomato, green pepper, onion, maize and the like from the Gedeb river. They produce these commercial food crops using water pump generators. Thus, if the irrigation dam site is selected, the local community will benefit at large. In addition, in and around the proposed irrigation dam site, there are no cultural heritage sites which will be removed. Thus, the target area has no such a tangible side effect on social and cultural aspects and it meets the irrigation dam site demands.

##### 2.3.1.4 Economic viability

**Construction costs:** the site should be economically feasible, considering factors such as accessibility, construction materials, and labour availability [77].The proposed irrigation dam site approximately has 3 km from the international highway.The local community has built up from productive and young age groups and thus, in the proposed irrigation dam site there is the best labour availability. In addition, the proposed irrigation dam site will increase potential agricultural productivity and water supply for the local community. Thus, the proposed irrigation dam site will outweigh the costs even though the total construction cost has never been studied and it meets the irrigation dam site demands.

##### 2.3.1.5 GCP and ASTER DEM

**Fig. 6:**
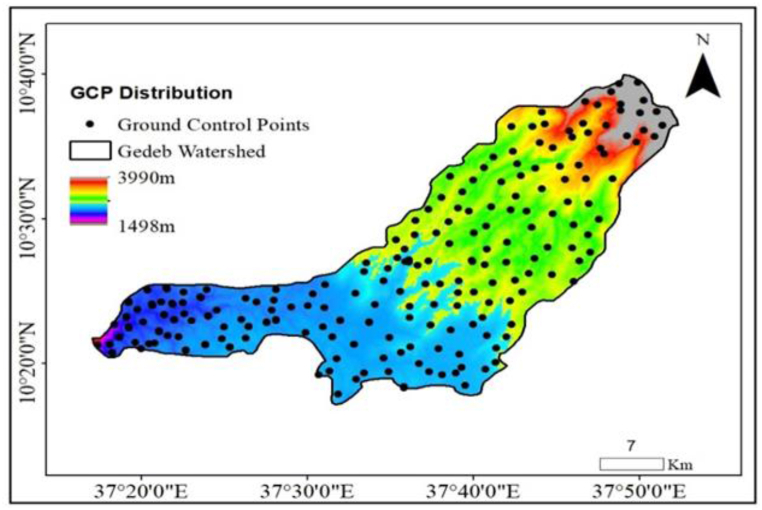
Spatial distribution of the collected GCPs from the Gedeb watershed.

To estimate suitable irrigation dam site selection and potential area in the Gedeb River, ASTER DEM data was used. To use the data confidentially, data quality measurements were taken on the data and were well distributed (Fig. 6). Random 223 Ground Control Points (GCP) were collected directly from the field using a Differential Ground Position System (DGPS).In addition, 223 GCP fitted values were extracted from the DEM using the extracted value to points arc tool. The minimum GCP and DEM point values were 1659m and 1658m, but the maximum values were 3931m and 3930m, respectively.

###### 2.3.1.5.1 GCP and ASTER DEM data validation

**Fig. 7:**
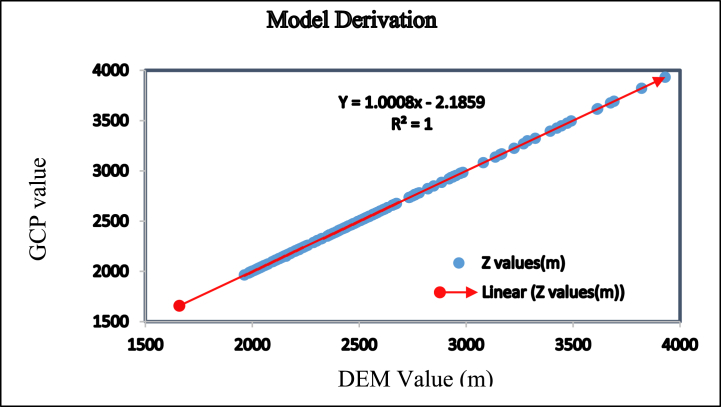
Relationship between the ground collected and the DEM extracted GCP points.

**Cross-validation** refers to a set of methods for measuring the performance of a given predictive model on new test data sets [78,79]. Thus, to cross-validate the goodness fit of the altitude values for both ground control points and the DEM points, 223 fitted points were used. The minimum GCP and DEM point values were 1659m and 1658m, but the maximum values were 3931m and 3930m, respectively. This implies that there is a 1m deviation between the minimum and maximum altitude values in the data. Although the data ranges in both data are large, the deviation is 1m. Thus, it is possible to conclude that there is no race variation in the data. In addition, to cross-validate the data, R-squared (R^2^); represents the squared correlation between the GCP values and the DEM values by the model and Mean Square Error (RSME); which measures the average difference between the observed known outcome values and the values predicted by the model [80].Thus, to drive them, a linear model was derived from the existing data and using the model, both R^2^ and RMSE of the data were calculated. Accordingly, the RMSE and R^2^ of the data become 0.9527m and 1m, respectively (Table 2 and Fig. 7). The higher the R^2^ and the lower the RMSE, the better the model [81]. Generally, within this large data range, a 1m difference is quite tolerable and valid to use the data for the study.

**Table 2:** Data Cross-validation using R.

Items(m)	No. points	Minimum	Maximum	Mean	Median	Standard Deviation
GCP Z Values	223	1659	3931	2415	2301	403.9917
DEM Z values	223	1658	3930	2415	2300	404.3026
Derived Model	223	1658	3932	2415	2301	404.3149
Residuals Result	223	−8.5413	10.5563	−0.0871	−0.4293	1.35733
Square Residuals	223	0.000	111.4355	1.8417	0.3454	8.98473
RMSE Result	223	0.01604	1.35708	***0.9527***	1.07044	0.371872

#### 2.3.2 Land suitability factors for irrigation area identification

Land suitability categorization is an approach to classifying a unique area of land considering its conduciveness for unique uses [64]. This system of land suitability evaluation is decisive for supplying crucial information about lots of restrictions and possible prospects for land uses that are being studied considering land competences [64], and they can be identified based on the selected parameters. For this study, cover/land use, Altitude, soil, distance, slope, geology, and land parameters were used to show the suitable area for irrigation.

##### 2.3.2.1 Structure of the land suitability classification for irrigation area identification

According to FAO [82,83], as described by Yonas et al. [64], land suitability classes are described as the extent of suitability for a specific kind of landscape for unique use, and its proposed approach for assessing landscape suitability in consideration of conducive scale ranging from highly suitable (S1), moderately suitable (S2), marginally suitable (S3), and to not suitable S4 (N1) considering the conduciveness of topographic properties for different crops type (Table 3).

**Table 3:** Land suitability classification.

FAO symbol	Suitability	Description	Land Index
Class 1	Highly	•Land with no appreciable restrictions (sustained application)	75–100
Class2	Moderately	•Land having limitations (moderately severe for sustained application)	50–75
Class 3	Marginally	•Land having limitations (aggregate are severe for sustained application)	25–50
N	Not	•Land that appears to prevent sustained use of under-consideration	0–25

##### 2.3.2.2 Land suitability parametrs assignment for irrigation area identification

**Fig. 8:**
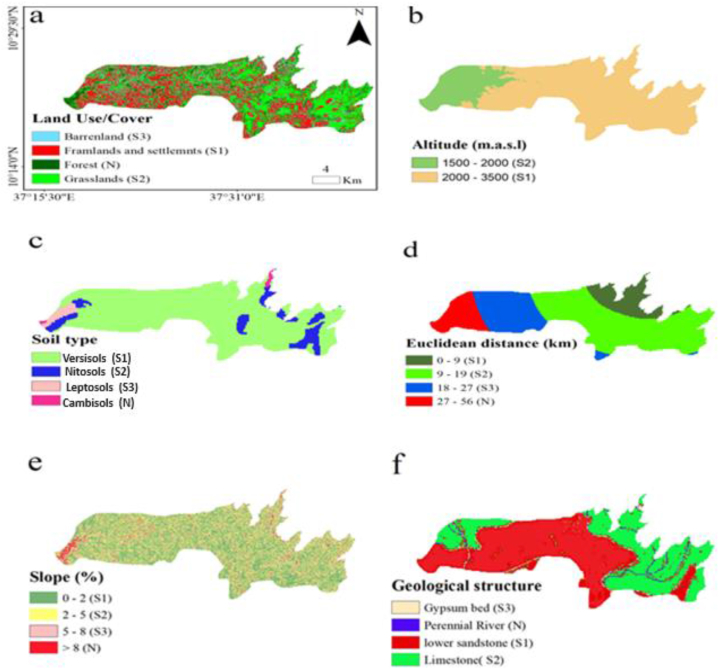
Parameters used for Irrigation Area suitability analysis.

Land use land cover (LULC) was an important factor used to identify the suitability land classification from the SPOT image and to classify the major classes; expert judgment and Spectral Angle Mapper were used to classify the essential land-use types that existed in the target area and were categories as farmland/settlement (S1), grassland (S2), barren (S3) and forest land (N) (Fig. 8a). In addition, Altitude was one of the parameters taken into consideration and divided into two classes: 1500–2000m (S1) and 2000–3500m (S2) (Fig. 8b). Similarly, the soil had a significant role in defining an area's potential for agricultural and long-term irrigation [84]. The soil's irrigation suitability was estimated using the revised Food and Agricultural Organization (FAO) soil map [64,85,86]. Furthermore,FAO [85] divides soil drainage into four categories: well, moderately well, imperfectly well, and poorly drained. Thus, based on their drainage capacities, the soil types in the target area were classified as Vertisols (S1), Nitosols (S2), Leptosols (S3), and Cambisols (N), which influenced the evaluation (Fig. 8c). Similarly, water source distance is one of the essential standards for defining land suitability for irrigation [16,64]. The proximity of the land to the water source was classified into four classes based on the equal interval (9 km) between the classes using Euclidian distance using ArcGIS (Fig. 8d). Furthermore, land suitability was assessed using the target area's topographic characteristic, which has a direct impact on irrigation. The slope was computed using a 20 m resolution DEM and classified into four groups based on the Mandal et al. [13], and Yonas et al. [64] classification systems: 1–2% slope is 95 % suitable, 2–5% slope is 90 % suitable, 5–8% slope is 80 % suitable, and more than 8 % slope is 70 % suitable for none terraced slopes. In addition, Mandal et al. [13] and Yonas et al. [64] assigned high weights (S1 class) to areas with slopes between 0 and 2 % because they perfectly percolate water to the crop root horizon, whereas percentages greater than 8 % were considered unconducive because they tend to runoff rather than percolate to the ground (Fig. 8e). Finally, a geological structure is one of the fundamental aspects in assessing the agricultural adaptability of the land, and it was classified as lower sandstone (S1), limestone (S2), gypsum bed (S3), and perennial river (N) (Fig. 8f).

##### 2.3.2.3 Weight of land suitability parameters for irrigation area identification

For land suitability analysis, Altitude, slope, land use land cover, soil, geology, and the distance from the water source were given their weight (Table 4). The type of LULC is the determinant factor that influences the suitability classification, and it was assigned 40 % weight in the suitability analysis. It was categorized as farmland/settlement (S1), grassland (S2), barren land (S3) and forest (N) with 13639.36ha (34.39 %), 14822.6ha (37.37 %), 6147.6ha (15.5 %), and 85054.68 (1274 %) spatial coverage respectively. Thus, the analysis assigned farmland/settlements and forests a high and low relative influential value, respectively. In addition, Altitude is one of the influential factors that determine irrigation suitability classification, and it was represented by an 11 % influential weight in the evaluation. Fortunately, the target area was grouped into 1500–2000 (S2) and 2000–3500 m.a.s.l (S1) altitude ranges based on the land suitability classification rate with 7549.3ha (19.03 %) and 32114.94ha (80.97 %) area coverage respectively. Moreover, the soil is one of the crucial factors that determine the irrigation suitability of analysis. Taking into account its availability, it was assigned by 19 % influential weight in the assessment. The soil types in the target area were Vertisols (with 34380.89ha (86.68 %)), Nitosols (4040.037ha (10.19 %)), Leptosols (970.8841ha (2.448 %)) and Cambisols (8.959ha (0.687 %)) spatial extent respectively. Thus, a high weight was given to Vertisols while a relatively low weight was given to Cambisols in the analysis. Likewise, land situated closer to water sources was considered more suitable for surface irrigation [64]. Generally, weights are allotted based on the feature's characteristics and its relationship to irrigation suitability [64]. The proximity to the water source was assigned by a 6 % impact load in the assessment. Therefore, the proximity of the land to the water source was classified into 0–9 km, 9–18 km, 18–27 km, and 27–36 km. Thus, high weight was given to the nearest area while low relative influential weight was assigned to distant areas. Furthermore, In addition, the slope is one of the influential parameters for the determination of any suitability analysis. The target was classified into 0–2 % (S1), 2–5 % (S2), 5–8 % (S3) and >8 % (N) with 19564.68ha (49.33 %), 13393.64ha (33.77 %), 5121.88ha (12.91 %) and 1584.04ha (3.994 %) area coverage respectively. So, even slope areas were allotted a high comparative effect value, while steep slope areas were assigned a low relative influence value in the weight overlay computation. Finally, geology had its impact on irrigation suitability determination, and the target area was categorized into four classes lower sandstone, limestone, Gypsum bed, and Perennial river with 12762.51ha (32.2 %), 22132.42ha (55.82 %), 1400.334ha (3.53 %) 3368.977ha (8.49 %) area coverage respectively (Table 4).

**Table 4:** Factors for surface irrigation land suitability assessment.

Parameters	Classes	Suitability	Weight	Area (ha)	Area (%)
LULC	Farmland/settlement	*S1*		13639.36	34.39
	Grassland	*S2*	40	14822.6	37.37
	Barren land	*S3*		6147.6	15.5
	Forest	*N*		5054.68	12.74
Altitude(m)	2000–3500	*S1*		32114.94	80.97
	1500–2000	*S2*	11	7549.3	19.03
Soil	Vertisols	*S1*		34380.89	86.68
	Nitosols	*S2*	19	4040.037	10.19
	Leptosols	*S3*		970.8841	2.448
	Cambisols	*N*		8.959	0.687
Distance(km)	0–9	*S1*		5238.479	13.2
	9–18	*S2*	6	21095.73	53.17
	18–27	*S3*		8741.198	22.03
	>27	*N*		4597.496	11.59
Slope (%)	0–2	*S1*		19564.68	49.33
	2–5	*S2*	20	13393.64	33.77
	5–8	*S3*		5121.88	12.91
	>8	*N*		1584.04	3.994
	Lower sandstone	*S1*		12762.51	32.2
Geology	Limestone	*S2*	4	22132.42	55.82
	Gypsum bed	*S3*		1400.334	3.53
	Perennial river	N		3368.977	8.49

##### 2.3.2.4 Weight assignment using the analytical hierarchy process (AHP)

Each sharing factor in the AHP was assigned a weight. It is the step of identifying criteria to measure the situation of decisions in spatial context [64,[87], [88], [89]]. It distributes weights using three processses:decomposition, comparison judgment, and priority synthesis [64]. It was employed in the multi-criteria decision-making strategy, which generates a matrix of pairwise comparisons between the characteristics that influence land suitability for irrigation [64,90]. AHP uses a scale of 1–9 to indicate if the two characteristics are equally important or if one is more important than the other. Reciprocal cross of one to nine (1/1 and 1/9) show that one is less essential than the other (Table 5). To get the eigenvalues, which indicate parameter weights, a pairwise comparison of contributing components was conducted and standardized. The random consistency indices (R.I.) were reported by Pant et al. [91] and Yonas et al. [64], respectively, were used to calculate the consistency ratio (C.R.), which confirms the level of consistency.

**Table 5:** Saat's random consistency indices in AHP [92].

Intensity	Definition	Explanation
1	Equal Importance	Two elements contribute equally to the purpose.
3	Moderate importance	Experience and judgment slightly favour one ingredient over another.
5	Strong importance	Experience and judgment favour one ingredient over another.
7	Very strong importance	One element is significantly favoured over another; its dominance may be shown in practice.
9	Extreme importance	The evidence that favours one element over another is of the greatest grade of affirmation.

2,4,6 and 8 can be used to express intermediate values.

The consistency index (CI) is calculated using the following formula:

$$CI=\frac{\lambda_{\max}-n}{RI}$$

• *λ*_max_ is the highest eigenvalue of the pairwise comparison matrix
• n is the number of classes.

Then, C.R. is given by the following formula [92]:

$$CR=\frac{CI}{RI}$$

Where RI is the ratio index/average value of CI for random matrices using the Saaty scale, and the weights of the decisive thematic maps were calculated using multi-criteria decision analysis. Expert knowledge and a survey of related literature were utilized to weigh irrigation suitability estimation parameters [64,93]. The AHP approach was used to calculate the normalized weights of the various themes and parameters. The pairings of criteria in the row and column were evaluated using the following criteria: first, which principle was more important, and second, how much the criterion above is more significant relative to the less important standard. The normalized weights were calculated by dividing each row result by the sum of the column results. The consistency ratio (C.R.) was determined to be 4 %, which is less than 10 % and indicates an acceptable level of consistency in the pairwise comparison (Table 6). The AHP approach demonstrated reasonable accuracy and can be used to estimate irrigation suitability.

**Table 6:** Pairwise comparison matrix for assessing the relative relevance of six factors.

Matrix							Normalized
							Principal
	LULC	Altitude	Soil	Distance	Slope	Geology	Eigenvector (%)
LULC	1	6	2	6	2	9	39.64
Altitude	0.17	1	0.5	3	0.5	4	10.93
Soil	0.5	2	1	6	1	2	19.10
Distance	0.17	0.33	0.17	1	0.2	3	5.49
Slope	0.5	2	1	5	1	6	20.78
Geology	0.11	0.25	0.5	0.33	0.17	1	4.06
CR = 4 % Consistency is	accepted						

### 2.4 Methodology workflow of the study

**Fig. 9:**
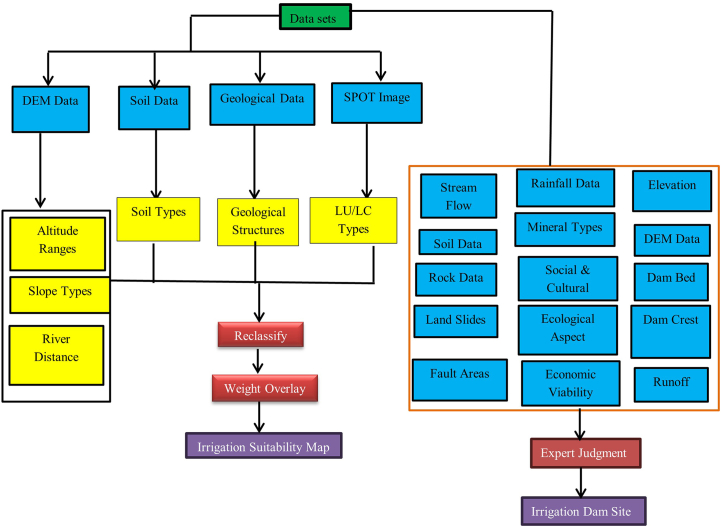
General methodology workflow of Irrigation damsite selection.

To accomplish the intended objective DEM, soil, geological, and SPOT image datasets were used. The necessary altitude, slope, and distance parameters were derived from the DEM data. In addition, soil type, geological structure, and LU/LC type data were generated from the soil, geologic, and SPOT image data respectively. Moreover, to make ready the parameters for overlay analysis for land suitability classification, those parameters were again reclassified. Finally, integrating the AHP calculator and weighted overlay analysis, the potential suitability areas were identified (Fig. 9).

## 3 Results

### 3.1 Possible irrigation dam site and suitable irrigation area

**Fig. 10:**
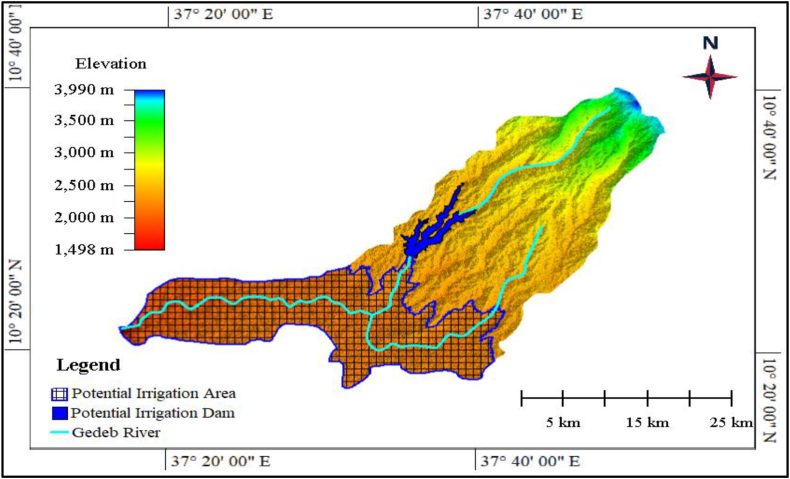
Potential irrigation dam and area in the Gedeb watershed.

A possible water reservoir area lies between 1155742.12 mN and 1166702.12 mN to 345666.89 mE-354137.17 mE, and this water reservoir area has 157 m in height from the river bed and 1.3 km dam axe length, respectively. Moreover, the south to northern extent of the potential water reservoir area covers 10.96 km, while the west to eastern extent covers 84.71 km. Thus, from this extent, it is possible to conclude that the west to eastern extent of the potential water reservoir area is by far larger than the north-south extent of the water reservoir area in which the water will lay. Therefore, more amount of reserve water potential will lay towards from west to east extent, and more water depth and much cubic water will accumulate in southwest parts of the reservoir, but a small amount of water level will be found in the North West direction of the water reservoir area, and it has a total area of 1886ha and potential of holding 2,961,145,697 m^3^ water and this amount of water will have a capacity of watering 39664.24ha of an irrigable agricultural field to the down face of the potential dam (Fig. 10).

### 3.2 Irrigation land suitability classification

**Fig. 11:**
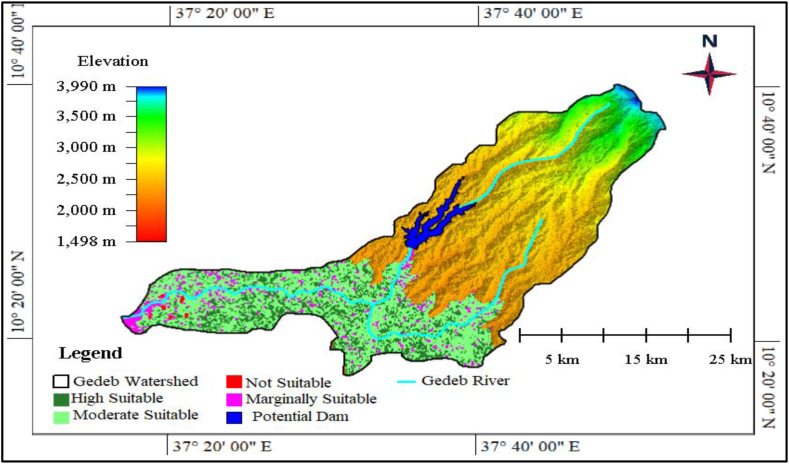
Land suitability classes for potential irrigation in the Gedeb watershed.

After the potential irrigation dam site had identified from the Gedeb watershed, the possible agricultural irrigable area determined and covered 39664.24ha. Thus, to determine the suitability of irrigable area, the identified potential irrigable area was classified into the high suitable, moderately suitable, marginally suitable, and not suitable area, major suitability classes. Spatially, the high suitable was located dominantly in the central parts, majorly covered by farmland and settlements, of the potential irrigation area, and it covered 18362.05ha (46.29 %) of the 39664.24ha. In addition, the moderate suitable area dominantly located in the south and southeast parts of the potential irrigable area and specifically was covered by grasslands. It covered 19204.05ha (48.42 %) of the total area. Similarly, the marginally suitable were located in the north and extreme southern parts of the potential irrigation area and these areas were known for their steep slope. It accounted for 2095.25ha (5.282 %) of the total potential area. Finally, the not suitable was precisely occupied by forests and hill peaks and found in the rear parts of the potential irrigation area; it covered 2.89ha (0.007 %) of the suitable area (Fig. 11).

## 4 Discussions

### 4.1 Possible irrigation dam site selection

Irrigation dams are essential for water management, agricultural development, flood control and ensuring water availability for various sectors [[1], [2], [3], [4]]. Taking this idea as a spring board, the study tried to investigate possible irrigation dam site selection in Gedeb watershed using historical rainfall, historical runoff, historical stream flow, fauling areas, landslide location, rock types, elevation, soil types, mineral types, geology, dam crest GCP and river bed GCP datasets (Table 1) of the target area using expert judgement decision making method. Based on this, proposed irrigation dam site was identified with a total area of 1886ha and potential of holding 2,961,145,697 m^3^ water and this amount of water will have a capacity of watering 39664.24ha of an irrigable agricultural field to the down face of the potential dam (Fig. 10).

The study on the selection of dam sites used geographic information systems and remote sensing techniques in conjunction with AHP and the selection of multiple criteria [94]. Data from the Shuttle Radar Topography Mission (SRTM) 30 m, LANDSAT-8 picture, soil data, and geology data were used in this investigation. Four categories—highly suited, somewhat suitable, least suitable, and not suitable—are shown by the study's findings. In addition, the study carried out on dam site selection using an integrated method of AHP and GIS for decision making support in Bortala, Northwest China [66] with Perception, slope, geology layer, soil type, land cover and drainage order datasets. Eight potential dam locations in Bortala with comparatively high suitability were suggested after a suitability map for the project was created. West Bortala, with its comparatively high elevation, was found to be the densest area with high and very high suitability for building a dam, while NW and Central Bortala were found to have low or extremely low suitability. This information is based on the suitability map. It was created to provide a profile of the proposed dams that included cross-sectional features including height, width, and volume. Two tiny dams, five medium-sized dams, and one large dam with a storage capacity ranging from 122,506 m^3^ to 5,033,652 m^3^ were among the proposed dams. Our study's results are not consistent with the methodologies and conclusions of these two studies.

### 4.2 Irrigation land suitability classification

Irrigation suitability analysis is important for optimal water management, crop selection and productivity, water conservation, environmental sustainability, and economic viability [12]. It provides valuable insights that enable stakeholders to make informed decisions and ensure the sustainable and efficient use of water resources in agriculture [13]. This concept inspired us to conduct irrigation suitability analysis in the bottom parts of Gedeb watershed using slope, land use land cover change, soil type, geological structure, altitude and distance from water source datasets of the target area using multi-criteria decision making method. Based on this, high suitable, moderately suitable, marginally suitable, and not suitable irrigation area were identified (Fig. 11).

The study carried out in the Shali River basin area, West Bengal, India, using the geographic information system and analytical hierarchy process [19] with elevation, slope, rainfall, soil group, sand, silt, clay, nitrogen, pH, organic carbon, land use and land cover, and river distance determined factors to identify appropriate irrigation land.The findings indicate that about 64 % of the land in the target area is eligible for irrigation which is also agreeable statement in case of our study.

Interestingly a research was carried out in West Shewa zone of Oromia, Ethiopia on evaluating land suitability for irrigation development [95] using an analytical hierarchy technique based on geographic information systems with Key elements including soil, slope, land use/cover, proximity to roads, rivers, urban areas, and rainfall. The finding categorized the agricultural lands in the region from extremely good to permanently unsuited for irrigation. 10.27 % (1419.87 km 2) of the total were highly suitable, 73.23 % (10,128.97 km 2) were fairly acceptable, 16.34 % (2259.95 km 2) were marginally suitable, and 0.16 % (22.16 km 2) were not suitable which is also agreeable statement in case of our findings.

Likewise the study carried out on assessing Sudan's potential for irrigated agriculture to project future increases in water pressure in the area. Geographic information systems (GIS) develops a model for land suitability analysis [96]. The study was conducted using by temperature, precipitation, slope, land cover, and specific soil qualities datasets. The land suitability analysis generating maps identify regions that are ideal for irrigated agriculture and those that are not which is also agreeable statement in case of our findings.

Similarly, the study carried out on finding suitable irrigation lands for the Minch Yekest watershed in West Amhara, Ethiopia [97]. The study used Slope, land usage, height, distance from the water source, soil properties, and available water storage capacity were taken into consideration with applied multicriteria and geographical decision-making approaches. Based on guidelines from the Food and Agricultural Organization, the irrigation land suitability of each physical land attribute was divided into four suitability classes (S1, S2, S3, and N). The results show that, 63 % of the watershed area is extremely suitable, 6.25 % is moderately acceptable, 28.69 % is marginally suitable, and 2.06 % is not suitable which is also agreeable statement in case of our method and investigations.

In addition, the study carried out on assessing the suitable land for surface irrigation using normalized difference vegetation index, soil, slope, proximity, rainfall deficit, and land use spatial parameters [98]. A weighted overlay tool was used to carry out the overlay suitability analysis. Thus, based on finding, Only 28.46 % of the basin's total land area was eligible for surface irrigation. Likely, the study carried out on irrigation site selection using multi-criteria decision analysis approach to analyze datasets such as slopes, rivers, land use, soil types, soil depths, water quality, water quantity and drainage patterns [99]. The study found that in Kasungu district, 36.9 % of the land is highly suitable, 20.7 % is moderately suitable, 33.1 % is lowly suitable and 9.3 % is not suitable for irrigation which are also agreeable statements in case of our method and investigations.

Moreover, the study carried out on evaluating the suitable land resource potential for irrigation development for the Katar River watershed in the Rift Valley Basin in Ethiopia by using ArcGIS based on Multi-Criteria Evaluation techniques [100]. The study used suitability factors, including land cover and use, proximity to rivers and cities, drainage classes, slope, soil texture, and depth. Thus, according to the weighting analysis of all characteristics, 34.08 % was categorized as very suitable (S1), 58.08 % as moderately suitable (S2), 3.8 % as marginally suitable (S3), and 3.21 % as not suitable (N) for surface irrigation development which is also agreeable statement in case of our method and investigations.

Incontrast, the study carried out on determining whether the area's soil qualities were suitable for irrigation with soil characteristics [101]. The parametric method was utilized to conduct the qualitative evaluation. The majority of the cultivated area (308.1ha), according to the results, is considered moderately appropriate (S3) land because of its medium soil depth and moderate slope. Because of the area's moderate slope, gravelly soil texture, and medium soil depth, 33 ha are considered unsuitable (N1). Additionally, 120.2ha in the research region was determined to be permanently unsuitable (N2) due to the high slope, gravelly soil texture and shallow soil depth which is contrast statement in case of our decision making proccess.

## 5 Conclusions and recommendations

This study aimed to identify a suitable dam site and irrigation area in the Gedeb River, Ethiopia, using 3D visualization methods. The research utilized various factors such as precipitation, water flow, geological formations, fault lines, landslide-prone areas, rock types, terrain features, soil types, elevation, slope, distance, and land cover information to determine an appropriate location for a dam and irrigation zone. The study employed remote sensing techniques, geographical information systems, multi-criteria decision-making methods and expert judgment decision-making methods.

The findings revealed a suitable irrigation water storage dam covering 1886 ha, with a potential water storage volume of 2,961,145,697 cubic meters. Additionally, the study identified a highly suitable region spanning 18,362.05 ha, a moderately suitable region covering 19,204.05 ha, a marginally suitable area totaling 2095.25 ha, and an unsuitable area of 2.89 ha for irrigation purposes.

The study presents research findings and methodology that can help government and non-governmental organizations plan irrigation projects effectively. Using 3D visualization, expert opinions, and multi-criteria decision-making methods, the study offers insights into water management, crop selection, water conservation, environmental sustainability, and economic feasibility in agriculture. It guides the selection of an appropriate location for a dam and irrigation zone in the Gedeb River area of Ethiopia, promoting sustainable farming practices. Further research is needed to validate the findings, considering socio-economic factors and stakeholder consultations. A thorough feasibility study on the proposed irrigation dam site, including engineering assessments and cost-benefit analysis, is crucial for project viability. Collaboration among government agencies, local communities, and experts is important for informed decision-making. Regular monitoring and maintenance of the dam site are necessary for long-term sustainability. Stakeholder involvement in decision-making is key to addressing their concerns.

## Funding declaration

The authors declare that there was no financial support from any funding agency, organization, or institution for this study.

## Data availability statement

The datasets generated during and/or analyzed the current study are available from the corresponding author upon reasonable request.

## CRediT authorship contribution statement

**Fekadu Temesgen:** Writing – review & editing, Writing – original draft, Visualization, Validation, Software, Methodology, Formal analysis, Data curation, Conceptualization. **Baye Terefe:** Writing – review & editing, Writing – original draft, Visualization, Methodology, Conceptualization.

## Declaration of competing interest

The authors declare that they have no known competing financial interests or personal relationships that could have appeared to influence the work reported in this paper.
